# Sociodemographic and workload-related determinants of cognitive perception among university faculty members: a cross-sectional survey study

**DOI:** 10.3389/fpsyg.2026.1866788

**Published:** 2026-09-25

**Authors:** Maryam N. ALNasser

**Affiliations:** Department of Biological Sciences, College of Science, King Faisal University, Al-Ahsa, Saudi Arabia

**Keywords:** academic environment, cognitive failures, cognitive perception, faculty members, occupational stress, sociodemographic factors, teaching load, workload

## Abstract

Cognitive failure describes everyday lapses in memory, attention, and task performance during routine activities. In academic settings, faculty members frequently manage demanding teaching loads, research commitments, and administrative duties, all of which may contribute to cognitive overload. Sociodemographic factors—such as age, and gender—may further shape how individuals manage these responsibilities and experience cognitive difficulties. A cross-sectional, self-report survey was conducted using the validated cognitive failures questionnaire (CFQ) to assess self-perceived everyday cognitive failures and examine their associations with sociodemographic characteristics and workload among 112 faculty members at the College of Science, King Faisal University. The findings indicated that subjective cognitive difficulties were common, with nearly half of the participants reporting moderate cognitive difficulties and a considerable proportion reporting severe perceived cognitive difficulties. Female gender emerged as a significant independent predictor of greater self-perceived cognitive difficulties. A high teaching workload, particularly 31–40 teaching hours per week, was also independently associated with increased self-reported cognitive failures. In contrast, greater involvement in research activities and moderate administrative responsibilities were independently associated with lower levels of self-perceived cognitive difficulties, suggesting an inverse association. Although academic rank and years of experience were associated with self-perceived cognitive difficulties in unadjusted analyses, these associations were no longer significant after multivariable adjustment. These findings underscore the potential importance of balanced workload management for supporting cognitive health and faculty well-being; however, because this study relied on self-report measures within a cross-sectional design, prospective studies incorporating objective cognitive assessments are needed to confirm these associations.

## 1 Introduction

Cognitive performance is crucial for success in academic and professional environments. This is especially important for university faculty members, who frequently manage tasks that need focus, memory, decision-making, and problem-solving. Lapses in these domains, often called cognitive failures, can greatly affect productivity, lower teaching quality, and impact the accuracy and reliability of research (Pace et al., 2021). Cognitive failures are everyday errors in perception, memory, and motor skills that can happen even in healthy individuals, often because of internal or external stress (Bufano et al., 2024; Carrigan and Barkus, 2016).

Several factors can negatively influence cognitive functioning in daily life. Chronic stress has been suggested to influence brain regions involved in cognitive functioning, including the hippocampus, and may contribute to difficulties in memory and cognitive performance (Bufano et al., 2024; Girotti et al., 2024). Sleep problems, including insomnia and even short-term sleep deprivation, can reduce attention, memory, and overall cognitive performance (Bufano et al., 2024; Hyndych et al., 2025). Sedentary behavior has also been associated with poorer cognitive outcomes (Bufano et al., 2024; Gogniat et al., 2025). Conversely, protective factors such as regular physical activity, social engagement, and participation in mentally stimulating activities—along with cognitively demanding work—can help maintain and enhance cognitive abilities (Bufano et al., 2024; Dhahbi et al., 2025; Weaver and Jaeggi, 2021; Yang et al., 2025). The balance between these risk and protective factors, often shaped by occupational demands, plays a key role in preserving cognitive health or increasing vulnerability to impairment (Bufano et al., 2024; Yang et al., 2025; Zotcheva, 2025).

One of the main causes of psychological stress and cognitive overload in the context of higher education has been found to be faculty workload (Pace et al., 2021; Xu and Wang, 2023). Teaching, administrative work, student supervision, curriculum development, and research are just a few of the conflicting responsibilities that faculty members usually have to balance (Pace et al., 2021; Xu and Wang, 2023). Increased fatigue, decreased focus, and decreased cognitive efficiency have all been linked to high workload levels, which may increase the likelihood of cognitive errors (Bos et al., 2013; Bowling et al., 2015; Mahdavi et al., 2024). In scientific fields, where precision, coherence, and clarity are crucial, the consequences of such errors are particularly severe.

Additionally, sociodemographic factors such as age, gender, academic rank, and years of experience may influence an individual’s vulnerability to cognitive failures. For instance, age-related cognitive changes can affect memory and attention, while gender and marital status may be linked to differences in stress levels and coping mechanisms (Abramo et al., 2015; El-Sayed et al., 2025; Salthouse, 2010). Understanding how these factors interact with workload to affect cognitive functioning is essential for developing effective institutional strategies to support faculty well-being and performance.

Cognitive failures may involve several underlying cognitive processes, including those related to executive function (Goodhew and Edwards, 2025). Executive function refers to a group of higher-order cognitive abilities responsible for regulating attention, maintaining goals, monitoring performance, inhibiting inappropriate responses, and adapting behavior to changing demands (Diamond, 2013). These processes play an important role in managing complex tasks and supporting effective daily functioning. Difficulties in executive control may contribute to increased cognitive lapses, such as reduced attention, forgetfulness, and errors during routine activities. However, cognitive failures and executive function represent related but distinct concepts (Goodhew and Edwards, 2025). While executive function involves specific cognitive control abilities that are typically assessed using objective neuropsychological tests, cognitive failures reflect individuals’ self-perceived experiences of everyday cognitive difficulties. In the present study, executive function is considered a potential theoretical mechanism underlying cognitive failures; however, it was not directly measured.

Although cognitive difficulties related to occupational demands are recognized internationally, research on self-reported cognitive failures among university faculty in the Middle East, particularly Saudi Arabia, remains limited. Local research is important because cultural, institutional, and professional contexts may shape faculty workload and well-being. At King Faisal University, College of Sciences faculty are expected to meet demanding academic standards while managing teaching, research, and administrative responsibilities, yet little is known about how these demands relate to everyday cognitive functioning. This study addresses this gap by examining the independent associations between sociodemographic characteristics, specific workload domains, and self-reported cognitive failures using a multivariable analytical approach. By assessing teaching, research, and administrative responsibilities separately rather than treating workload as a single measure, the study provides a more detailed understanding of how different aspects of academic work relate to perceived cognitive difficulties. To our knowledge, this is the first study to examine these relationships among university faculty within this institutional and regional context, providing evidence that may inform workload management and faculty well-being initiatives.

## 2 Materials and methods

### 2.1 Study design and population

This research utilized a cross-sectional study design to investigate the correlation among sociodemographic factors, faculty workload, and self-reported cognitive failures. Specifically, the study investigated whether personal characteristics, including age, gender, and years of academic experience, as well as workload-related factors such as teaching, research, and administrative responsibilities, were associated with faculty members’ subjective cognitive perception at the College of Sciences, King Faisal University.

The research employed a self-report questionnaire alongside the Cognitive Failures Questionnaire (CFQ) to evaluate self-reported everyday cognitive failures and collect information regarding the faculty members’ workloads and sociodemographic variables.

The data were gathered through an online survey developed using a Google Forms template and disseminated via social media platforms, including WhatsApp and University email, to improve accessibility and promote participation. At the start of the questionnaire, participants were given a short summary of the research’s objectives and an invitation to take part. The participants’ completion of the online survey constituted consent, with the submission serving as implicit agreement.

The target population for this study involved all faculty members at the College of Sciences, King Faisal University. Eligible faculty members from different academic ranks (e.g., assistant professors, associate professors, and lecturers) and disciplines (e.g., physical and biological sciences) were invited to participate. Participation was voluntary, and all eligible faculty members who agreed to participate during the data collection period were included in the study.

To determine an appropriate sample size, Cochran’s Sample Size Formula was applied to ensure the results would be both statistically significant and generalizable. The calculation was based on the total number of faculty members (250), using a 95% confidence level and a 5% margin of error. This process yielded an estimated sample size of 152 participants, which was considered sufficient for meaningful analysis. However, only 112 complete questionnaires were ultimately received and included in the final analysis. The study participants comprised full-time academic staff representing a range of departments and academic ranks.

### 2.2 Questionnaire development

The primary instrument for data collection in this study was a self-report questionnaire, designed to gather information on the participants’ sociodemographic factors, workload, and self-reported everyday cognitive failures S1. Sociodemographic information was collected on participants’ personal characteristics, including age, gender, marital status, years of experience, academic rank, and nationality. These variables were used to assess how sociodemographic factors influenced the frequency and severity of perceived cognitive difficulties.

The workload section examined faculty members’ professional responsibilities, including the number of hours spent on teaching-related duties, research activities, and administrative tasks. Participants were also asked about their work-life balance, including how often they worked beyond regular office hours and how frequently they experienced stress or fatigue due to their workload. These data provided insights into how workload might have contributed to cognitive perception.

Cognitive failures were measured using the Cognitive Failures Questionnaire (CFQ), developed by Broadbent et al. (1982). The CFQ is a self-report questionnaire that captures an individual’s subjective perception of everyday cognitive slips (e.g., forgetting, losing concentration, misplacing items). This questionnaire was designed to assess cognitive deficits in three key areas: forgetfulness, distractibility, and false triggering. The CFQ contained 25 questions, covering four categories of cognitive failure: memory lapses, attentional lapses, action lapses, and perception lapses.

Each item was rated on a 5-point Likert scale ranging from “Never” to “Very Often.” The ratings of all 25 items were summed up to produce a total score ranging from 0 to 100, with higher scores indicating more frequent self-reported everyday cognitive failures. The calculated scores were categorized into three groups: normal (0–30), moderate (31–60), and severe (61–100). CFQ scores were analyzed both as a continuous variable and categorized into groups for descriptive presentation in the current study. The categories (0–30, 31–60, and 61–100) were defined by the researchers solely to facilitate interpretation of the distribution of self-reported cognitive failures within the study sample. These categories were not derived from validated clinical cut-offs and should not be interpreted as clinical classifications or indicators of cognitive impairment.

**Psychometric characteristics of the CFQ:** The CFQ is one of the most widely used self-report measures of everyday cognitive failures and has demonstrated satisfactory internal consistency, temporal stability and construct validity across non-clinical adult and occupational populations comparable to the present sample (Bridger et al., 2013; Broadbent et al., 1982; Carrigan and Barkus, 2016). Consistent with previous work, the 25 items were treated as a single summed dimension of self-perceived cognitive failure.

The questionnaire was administered in a bilingual format in which every CFQ item and its response options were presented in the original English wording together with an Arabic rendering, so that participants could respond in whichever language they preferred. The Arabic version was prepared through independent forward translations by two bilingual translators, followed by a review and reconciliation of the translations into one version. This version was then back-translated into English by an independent translator. The researcher reviewed any differences between the original and back-translated versions to ensure that the meaning and concepts remained consistent. The finalized Arabic version was then included in the pilot testing described above.

Internal consistency of the instrument in the present sample was quantified using Cronbach’s α with a 95% confidence interval, McDonald’s ω, the mean inter-item correlation, corrected item–total correlations and the change in α on deletion of each item; these results are reported in the Results section.

To ensure the workload section of the questionnaire was both reliable and relevant, a multi-step validation process was carried out. It started with face validation by three experts in the field, followed by pilot testing with a small group drawn from the target population. This helped identify areas that needed clarification or adjustment to better align with the study’s aims. Feedback from the pilot test informed several revisions.

### 2.3 Statistical analysis

Descriptive statistics were used to summarize participant characteristics, workload-related factors, and CFQ score and are presented as frequencies and percentages.

**Outcome variable.** The outcome was the CFQ total score, computed as the sum of all 25 items (possible range 0–100) and treated as an ordered categorical variable with three levels—normal (0–30), moderate (31–60) and severe (61–100)—as defined above. Because the outcome was ordinal rather than binary, associations were estimated using ordinal logistic regression fitted as a proportional-odds (cumulative logit) model. The odds ratios presented in Tables 1, 2 are therefore cumulative odds ratios and express the odds of being in a higher rather than a lower cognitive-failure category for a given predictor level relative to its reference level.

**Table 1:** Univariable ordinal logistic regression analysis for factors associated with self-reported cognitive failures (total score).

Predictor	Comparison (Reference)	β (SE)	Z	*p* value	Odds ratio (95% CI)
Gender	Female vs. Male	1.75 (0.46)	3.84	<0.001	5.74 (2.42–14.50)
Marital status	Divorced vs. Single	1.31 (1.09)	1.20	0.231	3.70 (0.45–37.10)
	Married vs. Single	0.68 (0.96)	0.71	0.477	1.98 (0.31–16.00)
	Widowed vs. Single	2.12 (1.40)	1.51	0.131	8.30 (0.54–150.90)
Academic rank	Lecturer vs. Teaching Assistant	−1.14 (0.86)	−1.32	0.187	0.32 (0.06–1.73)
	Assistant Professor vs. Teaching Assistant	−1.49 (0.76)	−1.96	0.049	0.22 (0.05–0.99)
	Associate Professor vs. Teaching Assistant	−0.86 (0.75)	−1.15	0.251	0.42 (0.09–1.84)
	Professor vs. Teaching Assistant	−2.51 (0.87)	−2.90	0.004	0.08 (0.01–0.43)
Years of teaching	6–10 vs. 1–5 years	0.79 (0.54)	1.46	0.144	2.20 (0.77–6.42)
	>10 vs. 1–5 years	0.73 (0.48)	1.51	0.130	2.07 (0.81–5.44)
Teaching workload	10–20 vs. < 10 h/week	0.87 (0.92)	0.95	0.345	2.39 (0.42–18.70)
	21–30 vs. < 10 h/week	1.18 (0.94)	1.26	0.208	3.26 (0.55–26.00)
	31–40 vs. < 10 h/week	2.11 (1.04)	2.02	0.043	8.22 (1.13–77.30)
	>40 vs. < 10 h/week	3.02 (1.61)	1.87	0.061	20.44 (0.90–738.20)
Research workload	5–10 vs. < 5 h/week	−1.49 (0.51)	−2.94	0.003	0.23 (0.08–0.60)
	11–20 vs. < 5 h/week	−1.20 (0.51)	−2.37	0.018	0.30 (0.11–0.80)
	21–30 vs. < 5 h/week	−1.57 (0.70)	−2.23	0.025	0.21 (0.05–0.81)
	>30 vs. < 5 h/week	−2.88 (1.22)	−2.37	0.018	0.06 (0.00–0.49)
Administrative workload	5–10 vs. < 5 h/week	−0.87 (0.44)	−2.01	0.045	0.42 (0.18–0.97)
	11–20 vs. < 5 h/week	−0.26 (0.54)	−0.49	0.628	0.77 (0.26–2.22)
	>20 vs. < 5 h/week	−1.93 (1.26)	−1.53	0.125	0.15 (0.01–1.48)
Working beyond office hours	Rarely vs. Never	0.61 (0.81)	0.76	0.449	1.84 (0.39–10.20)
	Often vs. Never	1.29 (0.77)	1.67	0.096	3.62 (0.83–18.90)

Values are presented as regression coefficient (β), standard error (SE), odds ratio (OR), and 95% confidence interval (CI). Reference categories are indicated in parentheses. *p* values <0.05 were considered statistically significant. Odds ratios are cumulative odds ratios from a proportional-odds (ordinal logistic) model and express the odds of being in a higher rather than a lower cognitive-failure category.

**Table 2:** Multivariable ordinal logistic regression analysis for factors associated with self-reported cognitive failures (total score).

Predictor	Comparison (Reference)	β (SE)	Z	*p* value	Adjusted odds ratio (95% CI)
Gender	Female vs. Male	1.49 (0.54)	2.74	0.006	4.43 (1.57–13.49)
Academic rank	Lecturer vs. Teaching Assistant	−0.56 (0.95)	−0.59	0.554	0.57 (0.09–3.66)
	Assistant Professor vs. Teaching Assistant	−0.64 (0.86)	−0.75	0.456	0.53 (0.09–2.84)
	Associate Professor vs. Teaching Assistant	0.50 (0.89)	0.56	0.577	1.65 (0.28–9.64)
	Professor vs. Teaching Assistant	−1.05 (1.06)	−0.98	0.325	0.35 (0.04–2.79)
Administrative workload	5–10 vs. < 5 h/week	−1.35 (0.51)	−2.66	0.008	0.26 (0.09–0.69)
	11–20 vs. < 5 h/week	−0.88 (0.69)	−1.28	0.201	0.41 (0.10–1.57)
	>20 vs. < 5 h/week	−0.57 (1.36)	−0.42	0.675	0.57 (0.02–6.98)
Teaching workload	10–20 vs. < 10 h/week	1.07 (1.10)	0.98	0.329	2.92 (0.38–31.14)
	21–30 vs. < 10 h/week	1.35 (1.13)	1.20	0.231	3.85 (0.47–43.45)
	31–40 vs. < 10 h/week	2.60 (1.23)	2.12	0.034	13.52 (1.36–185.26)
	>40 vs. < 10 h/week	2.86 (1.79)	1.60	0.110	17.37 (0.54–816.63)
Research workload	5–10 vs. < 5 h/week	−1.58 (0.61)	−2.60	0.009	0.21 (0.06–0.66)
	11–20 vs. < 5 h/week	−0.95 (0.64)	−1.48	0.139	0.39 (0.11–1.34)
	21–30 vs. < 5 h/week	−1.57 (0.84)	−1.88	0.060	0.21 (0.04–1.04)
	>30 vs. < 5 h/week	−2.33 (1.43)	−1.64	0.102	0.10 (0.00–1.34)

Values are presented as regression coefficient (β), standard error (SE), odds ratio (OR), and 95% confidence interval (CI). Reference categories are indicated in parentheses. *p* values <0.05 were considered statistically significant. Odds ratios are cumulative odds ratios from a proportional-odds (ordinal logistic) model and express the odds of being in a higher rather than a lower cognitive-failure category.

**Predictor variables.** All predictors were categorical and were entered as indicator (dummy) variables; none was modeled as a continuous variable. The predictors examined were gender, marital status, academic rank, years of employment, teaching workload, research workload, administrative workload and frequency of working beyond regular office hours. The reference categories were male gender, single marital status, teaching assistant, 1–5 years of employment, <10 teaching hours per week, <5 research hours per week, <5 administrative hours per week and never working beyond regular office hours; these are shown in parentheses in Tables 1, 2.

**Model building.** Each predictor was first examined in a separate univariable ordinal logistic regression model (Table 1). Variables for which at least one category reached *p* < 0.05 in univariable analysis—gender, academic rank, teaching workload, research workload and administrative workload—were then entered simultaneously into a single multivariable model (Table 2). Marital status, years of employment and frequency of working beyond regular office hours did not meet this threshold and were not carried forward. No automated stepwise procedure was used, and all categories of a retained variable were kept in the model irrespective of the significance of individual levels.

**Model assumptions and adequacy.** The proportional-odds assumption was examined using a likelihood-ratio test of parallel lines, comparing the fitted proportional-odds model with an equivalent model in which the coefficients were allowed to vary across outcome thresholds. The assumption was supported for the multivariable model (χ^2^ = 15.01, df = 16, *p* = 0.524) and for each predictor tested separately (all *p* > 0.10). Multicollinearity was assessed using variance inflation factors, all of which lay below the conventional cut-off of 10 (maximum 6.98, arising between indicator variables belonging to the same categorical predictor). Overall model adequacy was evaluated by a likelihood-ratio test against the intercept-only model and by pseudo-R^2^ statistics.

**Assessment of common method bias.** Because all study variables, including both workload and cognitive failures, were obtained from self-report instruments completed by the same respondents on a single occasion, the potential for common method bias was formally examined using Harman’s single-factor test (Podsakoff et al., 2003). All 32 self-reported items entering the analyses—the 25 CFQ items together with the teaching, research and administrative workload items, frequency of working beyond office hours, sleep duration, weekday exercise and work-related stress or fatigue—were submitted to an unrotated exploratory factor analysis. The proportion of total variance accounted for by the first and largest extracted factor was compared with the conventional 50% threshold, above which a single common method factor is regarded as a plausible explanation for the observed associations.

**Reliability.** Internal consistency of the CFQ was quantified using Cronbach’s α with a 95% confidence interval, McDonald’s ω derived from a single-factor congeneric model (McDonald, 1999), the mean inter-item correlation, corrected item–total correlations and α-if-item-deleted. Sampling adequacy for factor analysis was verified using the Kaiser–Meyer–Olkin measure and Bartlett’s test of sphericity. Internal consistency was not computed for the workload section, which consists of independent single-item indicators of distinct activities rather than a reflective scale.

Results are reported as odds ratios (ORs) with 95% confidence intervals (CIs). A two-sided *p* value <0.05 was considered statistically significant. All analyses were performed in Python version 3.12.3, (Python Software Foundation, Wilmington, DE, USA) using the statsmodels (version 0.14.6) pandas (version 3.0.2), NumPy (version 2.4.4) and SciPy (version 1.17.1) packages.

### 2.4 Ethical considerations

This research obtained ethical approval from the Institutional Review Board (IRB) at King Faisal University under Ref. No. KFU-REC-2025-MAR-ETHICS3105. Informed consent was obtained from all participants prior to their involvement in the study. The consent process clearly explained the voluntary nature of participation, the confidentiality of responses, and the participants’ right to withdraw at any time without consequence. All responses were anonymous, and data were securely stored to protect participant privacy. The study adhered to established ethical guidelines for research involving human subjects.

## 3 Results

### 3.1 Participant characteristics

The sociodemographic, academic, and workload characteristics of the study participants are summarized in Table 3. More than half of the participants were aged 41–50 years (55.4%), and the majority were female (72.3%). Most participants were married (84.8%) and had children (83.9%). Approximately half of the sample were Saudi nationals (48.2%).

**Table 3:** Sociodemographic, academic, and workload characteristics of the study participants (*N* = 112).

Characteristic	n (%)
Age group (years)	
21–30	1 (0.9)
31–40	22 (19.6)
41–50	62 (55.4)
≥51	27 (24.1)
Gender	
Female	81 (72.3)
Male	31 (27.7)
Marital status	
Married	95 (84.8)
Divorced	10 (8.9)
Single	4 (3.6)
Widowed	3 (2.7)
Have children	
Yes	94 (83.9)
No	18 (16.1)
Nationality	
Saudi	54 (48.2)
Non-Saudi	58 (51.8)
Academic rank	
Teaching Assistant	7 (6.2)
Lecturer	13 (11.6)
Assistant Professor	38 (33.9)
Associate Professor	38 (33.9)
Professor	16 (14.3)
Department	
Biology	48 (42.9)
Chemistry	18 (16.1)
Mathematics	27 (24.1)
Physics	19 (17.0)
Years of employment	
1–5 years	21 (18.8)
6–10 years	30 (26.8)
>10 years	61 (54.5)
Teaching workload (hours/week)	
<10	5 (4.5)
10–20	52 (46.4)
21–30	40 (35.7)
31–40	13 (11.6)
>40	2 (1.8)
Research workload (hours/week)	
<5	27 (24.1)
5–10	35 (31.2)
11–20	35 (31.2)
21–30	11 (9.8)
>30	4 (3.6)
Administrative workload (hours/week)	
<5	30 (26.8)
5–10	57 (50.9)
11–20	21 (18.8)
>20	4 (3.6)
Working beyond regular office hours	
Never	8 (7.1)
Rarely (<3 days/week)	33 (29.5)
Often (>3 days/week)	71 (63.4)
Average sleep duration	
<6 h	57 (50.9)
≥6 h	55 (49.1)
Weekday exercise	
Yes	47 (42.0)
No	65 (58.0)
Work-related stress or fatigue	
Never	3 (2.7)
Sometimes	74 (66.1)
Always	35 (31.2)

Data is presented as number (percentage). Percentages may not total 100% due to rounding.

Regarding academic characteristics, assistant professors and associate professors each constituted 33.9% of the sample, while professors represented 14.3%. More than half of the participants (54.5%) had been employed for over 10 years. Teaching workload was substantial, with 82.1% of participants reporting ≥10 teaching hours per week, and 63.4% reported frequently working beyond regular office hours. Nearly half of the participants slept less than 6 h per night (50.9%), and 58.0% reported not exercising on weekdays.

### 3.2 Distribution of self-reported cognitive deficits

Figure 1 presents the distribution of participants according to their levels of self-perceived cognitive difficulties based on CFQ scores. Nearly half of the participants (48%) reported moderate levels of self-reported cognitive failures, while 36% reported low levels of cognitive difficulties. Notably, 16% of participants reported high levels of self-reported cognitive failures, indicating a considerable prevalence of perceived cognitive difficulties within the study population.

**Figure 1:**
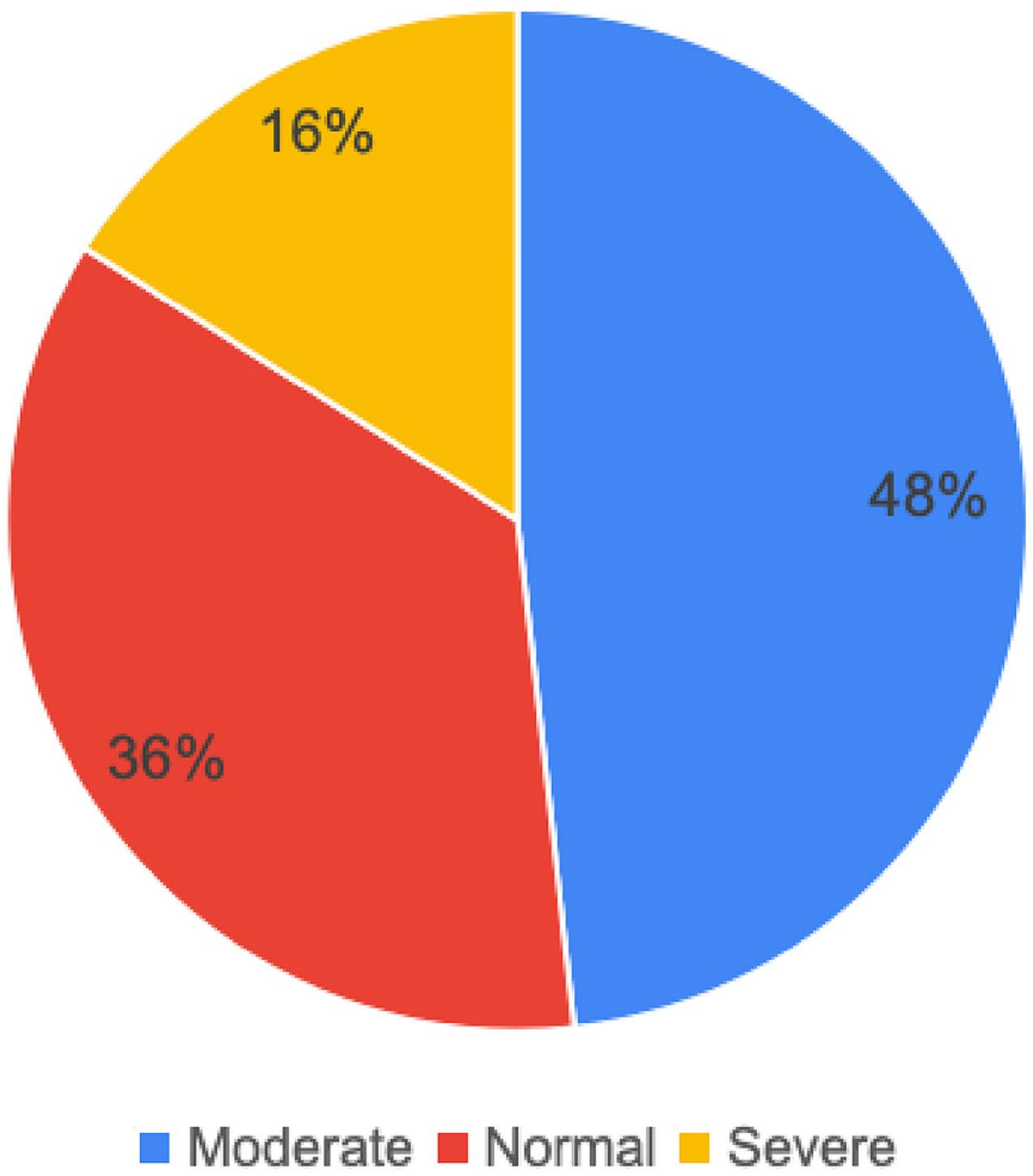
Distribution of self-perceived cognitive difficulties among study participants.

### 3.3 Univariable regression analysis

Results of the univariable ordinal logistic regression analysis are presented in Table 1. Female gender was significantly associated with higher odds of reporting greater levels of self-reported cognitive failures compared with males (OR = 5.74, 95% CI: 2.42–14.50). Higher academic rank was associated with lower odds of reporting elevated cognitive failure level, particularly among assistant professors (OR = 0.22, 95% CI: 0.05–0.99) and professors (OR = 0.08, 95% CI: 0.01–0.43), compared with teaching assistants. A higher teaching workload was associated with increased odds of greater self-reported cognitive failures, with a significant effect observed among participants reporting 31–40 teaching hours per week (OR = 8.22, 95% CI: 1.13–77.30). In contrast, greater involvement in research activities was consistently associated with lower odds of reporting cognitive failure levels, including 5–10 h per week (OR = 0.23, 95% CI: 0.08–0.60), 11–20 h per week (OR = 0.30, 95% CI: 0.11–0.80), and 21–30 h per week (OR = 0.21, 95% CI: 0.05–0.81), compared with less than 5 h. Additionally, administrative workload of 5–10 h per week was also associated with lower odds of reporting self-reported cognitive failures (OR = 0.42, 95% CI: 0.18–0.97).

### 3.4 Multivariable regression analysis

The multivariable ordinal logistic regression model is shown in Table 2. After adjustment for potential confounders, female gender remained an independent predictor of higher levels of self-reported cognitive failures (adjusted OR = 4.43, 95% CI: 1.57–13.49). A teaching workload of 31–40 h per week was independently associated with significantly increased odds of reporting higher levels of cognitive failures (adjusted OR = 13.52, 95% CI: 1.36–185.26). Conversely, moderate research workload of 5–10 h per week was independently associated with lower odds of self-reported cognitive deficits (adjusted OR = 0.21, 95% CI: 0.06–0.66), as was moderate administrative workload of 5–10 h per week (adjusted OR = 0.26, 95% CI: 0.09–0.69). Academic rank was no longer significantly associated with self-reported cognitive deficits after multivariable adjustment.

### 3.5 Reliability of the CFQ and assessment of common method Bias

Internal consistency of the 25-item CFQ was excellent in this sample (Cronbach’s α = 0.96, 95% CI 0.95–0.97; McDonald’s ω = 0.96). The mean inter-item correlation was 0.49 and corrected item–total correlations ranged from 0.40 to 0.84, with no item falling below the conventional 0.30 criterion. Deletion of no individual item produced a meaningful increase in α (range across items 0.957–0.961), and all 25 items were therefore retained. Sampling adequacy was excellent (Kaiser–Meyer–Olkin = 0.94) and Bartlett’s test of sphericity was significant (χ^2^(300) = 2003.9, *p* < 0.001), confirming that the item correlation matrix was suitable for factor analysis.

Harman’s single-factor test was performed on the 32 self-reported items entering the analyses. Unrotated exploratory factor analysis extracted seven factors with eigenvalues greater than 1, which together accounted for 68.8% of the total variance. The first and largest unrotated factor accounted for 42.1% of the total variance, below the conventional 50% threshold. Common method bias is therefore unlikely to provide a sufficient explanation for the associations reported above.

For the multivariable model, the proportional-odds assumption was supported (test of parallel lines: χ^2^ = 15.01, df = 16, *p* = 0.524), and the model fitted the data significantly better than the intercept-only model (likelihood-ratio χ^2^(16) = 46.70, *p* < 0.001; Nagelkerke R^2^ = 0.39). All variance inflation factors were below 10.

## 4 Discussion

This cross-sectional study examined the associations between sociodemographic, workload characteristics and self-reported cognitive failures among university faculty members in Saudi Arabia. Overall, self-reported cognitive failures were common, with almost half of participants reporting moderate cognitive difficulties and a substantial proportion reporting high levels of cognitive failures. Female gender and intensive teaching workload were independently associated with higher levels of perceived cognitive failures, whereas greater engagement in research activities and moderate administrative responsibilities was associated with lower odds of reporting cognitive failures. In contrast, academic rank and years of experience were not independently associated with cognitive failures after adjustment for other variables, suggesting that current occupational demands may be more closely related to subjective cognitive functioning than professional seniority alone.

These findings extend previous research on occupational stress and cognitive functioning by highlighting the cognitive demands of academic work. The results can be interpreted within the framework of Cognitive Load Theory, which proposes that working memory has limited capacity and that performance may be affected when cognitive demands exceed available resources (Sweller, 1988; Sweller et al., 2019). Faculty members routinely manage multiple cognitively demanding activities, including teaching, assessment, research, supervision, and administration. Such competing demands may increase cognitive load and contribute to everyday lapses in attention and memory. Importantly, the present study assessed subjective cognitive failures rather than objective cognitive impairment; therefore, these findings should be interpreted as reflecting perceived cognitive difficulties rather than evidence of neurological decline.

The observed association between teaching workload and cognitive failures is also consistent with theories of executive functioning. Executive processes, including working memory, attentional control, and cognitive flexibility, are essential for managing complex tasks and switching between competing demands (Diamond, 2013; Miyake et al., 2000). Intensive teaching responsibilities require continuous concentration, rapid decision-making, and frequent task switching, which may increase cognitive strain and awareness of everyday cognitive lapses. Similar relationships between high workload, mental fatigue, and reduced cognitive efficiency have been reported in occupational studies (Hopstaken et al., 2015; Van Cutsem et al., 2017). However, because of the cross-sectional design, the current findings cannot establish whether workload contributes to cognitive failures or whether individuals experiencing more cognitive difficulties perceive their workload differently.

The relationship between workload and cognitive functioning may also be understood through the Job Demands–Resources (JD-R) model, which suggests that high job demands can lead to strain when they exceed available personal and organizational resources (Bakker and Demerouti, 2017; Demerouti et al., 2001). In academic settings, excessive teaching responsibilities may represent a substantial cognitive demand requiring sustained attention, emotional regulation, and time investment. These findings suggest the importance of considering cognitive well-being as part of occupational health in universities, particularly in environments where faculty members face increasing teaching and administrative pressures.

Female faculty members reported higher levels of cognitive failures, consistent with previous studies reporting gender differences in cognitive complaints (Alshammari et al., 2020; Rahman and El Gaafary, 2009). The reasons for this association remain unclear but may involve differences in psychosocial demands, work–life balance, and exposure to multiple roles. Future studies incorporating measures of family responsibilities, burnout, and psychological stress are needed to better understand these differences.

In contrast, greater research engagement was associated with fewer self-reported cognitive failures. This may reflect the role of cognitive reserve and the “use-it-or-lose-it” hypothesis, which suggest that regular intellectual activity and mentally stimulating environments may help maintain cognitive resilience (Corbo et al., 2023; Hultsch et al., 1999; Kotliar et al., 2025; Salthouse, 2006; Salthouse, 2016; Stern, 2002; Stern et al., 2020). Research activities involve problem-solving, critical thinking, and continuous learning, which may help maintain cognitive engagement. Similarly, moderate administrative responsibilities may provide opportunities for autonomy, decision-making, and professional involvement. However, these findings should be interpreted cautiously, as the cross-sectional, self-reported nature of the study does not allow us to determine whether research and leadership activities improve cognitive functioning or whether individuals who perceive themselves as cognitively better are more likely to engage in these activities.

Finally, the finding that workload characteristics rather than academic rank or seniority, were more strongly associated with self-reported cognitive failures suggests that the immediate psychological and occupational demands of a role may be more relevant to subjective cognitive experiences than accumulated professional experience alone. This interpretation is supported by previous studies showing that high mental workload and occupational stress significantly correlate with cognitive failure regardless of the worker’s age (Allahyari et al., 2014; de Souza-Talarico et al., 2020; Kakemam et al., 2019).

Overall, the findings highlight cognitive well-being as an important aspect of faculty occupational health. Heavier teaching workloads were linked to more self-reported cognitive failures, suggesting the need for balanced workloads, adequate recovery time, and protected research time. Research engagement may support cognitive well-being through intellectually stimulating work. However, the cross-sectional, single-center design and reliance on self-reported measures limit causal conclusions and generalizability. Future longitudinal, multicenter studies using objective cognitive measures are needed to confirm these findings.

## 5 Study limitations

This study has several limitations that should be considered when interpreting the findings. First, the required sample size was estimated at 152 participants; however, the final sample included 112 faculty members. This difference was mainly related to the limited number of eligible participants, the voluntary nature of participation, and the predefined data collection period. To maximize the available data, all eligible faculty members who agreed to participate were included in the analysis. Although the sample size was sufficient to identify several significant associations, the smaller sample may have reduced the statistical power to detect smaller effects, meaning that some true relationships may not have reached statistical significance. In addition, because participation was voluntary, self-selection bias cannot be excluded, as faculty members who chose to participate may have differed from those who did not. Furthermore, as the participants were recruited from a single college at one university, the findings may not be fully generalizable to faculty members in other universities or academic settings.

Second, cognitive failures were assessed using the CFQ, which measures self-perceived everyday cognitive difficulties rather than objective cognitive performance. Therefore, the findings represent participants’ perceptions of their cognitive functioning and should not be interpreted as evidence of clinical cognitive impairment. Comparisons with previous studies should be made carefully, as differences in populations, research settings, and assessment methods may influence the findings.

Third, several factors that may influence cognitive functioning, including sleep quality, burnout, psychological stress, anxiety, depression, chronic health conditions, and work–life balance, were not assessed in this study. These factors may contribute to self-perceived cognitive difficulties and may influence the observed relationships between workload characteristics and cognitive failures. Therefore, residual confounding cannot be excluded, and the findings should be interpreted within the context of the variables measured. Future studies should include these psychological, health-related, and lifestyle factors to provide a more comprehensive assessment of the determinants of cognitive failures among faculty members.

Fourth, all study variables, including both workload and cognitive failures, were obtained from self-report questionnaires completed by the same respondents at a single point in time, which raises the possibility of common method bias. Harman’s single-factor test did not indicate a dominant common method factor, as the first unrotated factor accounted for 42.1% of the total variance, below the conventional 50% threshold. Harman’s test is nevertheless a relatively insensitive diagnostic and cannot exclude more subtle method effects; in particular, it cannot resolve the possibility that faculty members experiencing greater cognitive difficulty also appraise their workload as heavier. Future studies should separate the measurement of exposure and outcome in time, obtain workload data from institutional records rather than self-report, and complement subjective ratings with objective cognitive testing.

Despite these limitations, this study used a validated instrument, standardized data collection procedures, and multivariable analyses to account for potential confounding factors. To our knowledge, this is the first study to investigate the independent relationships between sociodemographic characteristics, specific workload domains (teaching, research, and administrative responsibilities), and self-reported cognitive failures among university faculty members in Saudi Arabia and the broader Middle Eastern academic context. Future studies involving larger, multicenter samples and longitudinal designs, with both subjective and objective measures of cognitive functioning, are recommended to confirm and expand upon these findings.

## 6 Conclusion

This study highlights self-reported cognitive failures as an important aspect of occupational well-being among university faculty in Saudi Arabia. Heavier teaching workloads and female gender were associated with higher levels of perceived cognitive failures, whereas greater research engagement and moderate administrative responsibilities were associated with fewer reported difficulties. By examining teaching, research, and administrative workload domains separately, this study contributes to the limited evidence on the associations between specific academic workload domains and self-reported cognitive failures among university faculty in Saudi Arabia and the broader Middle Eastern context. These findings suggest that current workload demands may be more relevant to subjective cognitive functioning than academic rank or years of experience. Universities should consider workload balance, adequate recovery time, and opportunities for meaningful research engagement as part of faculty well-being initiatives. However, the cross-sectional, single-center design and reliance on self-reported measures limit causal interpretation. Longitudinal, multicenter studies using objective cognitive assessments are needed to confirm these associations and clarify the factors influencing cognitive well-being among university faculty.

## Data Availability

The raw data supporting the conclusions of this article will be made available by the authors, without undue reservation.
