# Supplementary material for: Sociodemographic and workload-related determinants of cognitive perception among university faculty members: a cross-sectional survey study
Source: Front Psychol. 2026 Sep 25;17:1866788. doi: 10.3389/fpsyg.2026.1866788 (PMC13649409; doi:10.3389/fpsyg.2026.1866788)
Supplement: Supplementary file 1 [file Data_Sheet_1.DOCX]

Supplementary Material

# 1 Supplementary Data

S1. Study Questionnaire

Answer the following questions by circling the most appropriate answer.

**Sociodemographic Data**

1. **Age:**
   • 21-30 • 31-40 • 41-50 • 51 and above
2. **Gender:**
   • Male • Female
3. **Marital Status:**
   • Single • Married • Divorced • Widowed
4. **Do you have children?**
   • Yes • No
5. **Nationality:**
   • Saudi • Non-Saudi
6. **What is your current academic rank?**
   • Professor • Associate Professor • Assistant Professor • Lecturer • Teaching Assistant
7. **How long have you been employed as a faculty member at the College of Sciences, King Faisal University?**
   • Less than 1 year • 1-5 years • 6-10 years • More than 10 years

**Workload-related Data**

8. **How many hours per week do you spend on teaching-related duties (including lectures, grading, lab sessions, etc.)?**
   • Less than 10 hours • 10-20 hours • 21-30 hours • 31-40 hours • More than 40 hours
9. **On average, how many hours per week do you dedicate to research activities (including data collection, writing, reviewing, etc.)?**
   • Less than 5 hours • 5-10 hours • 11-20 hours • 21-30 hours • More than 30 hours
10. **How many hours per week do you spend on administrative tasks (e.g., meetings, committee work, student advising)?**
    • Less than 5 hours • 6-10 hours • 11-20 hours • More than 20 hours
11. **How often do you find yourself working beyond regular office hours (e.g., late evenings, weekends)?**
    • Often (More than 3 days per week) • Rarely (Less than 3 days per week) • Never (I do not work beyond regular office hours)
12. **How many hours of sleep do you get on average?**
    • Less than 6 hours • More than 6 hours
13. **Do you exercise on weekdays (Sunday-Thursday)?**
    • Never exercise • Exercise
14. **How often do you experience stress or fatigue due to your workload?**
    • Never • Sometimes • Always

**Cognitive Failures Assessment**

The following questions are about minor mistakes, which everyone makes from time to time, but some of which happen more often than others. We want to know how often these things have happened to you in the past 6 months. Please circle the appropriate number.

15. **Do you read something and find you haven’t been thinking about it and must read it again?**
    • 4 (Very often) • 3 (Quite often) • 2 (Occasionally) • 1 (Very rarely) • 0 (Never)
16. **Do you find you forget why you went from one part of the house to the other?**
    • 4 (Very often) • 3 (Quite often) • 2 (Occasionally) • 1 (Very rarely) • 0 (Never)
17. **Do you fail to notice signposts on the road?**
    • 4 (Very often) • 3 (Quite often) • 2 (Occasionally) • 1 (Very rarely) • 0 (Never)
18. **Do you find you confuse right and left when giving directions?**
    • 4 (Very often) • 3 (Quite often) • 2 (Occasionally) • 1 (Very rarely) • 0 (Never)
19. **Do you bump into people?**
    • 4 (Very often) • 3 (Quite often) • 2 (Occasionally) • 1 (Very rarely) • 0 (Never)
20. **Do you find you forget whether you’ve turned off a light or a fire or locked the door?**
    • 4 (Very often) • 3 (Quite often) • 2 (Occasionally) • 1 (Very rarely) • 0 (Never)
21. **Do you fail to listen to people’s names when you are meeting them?**
    • 4 (Very often) • 3 (Quite often) • 2 (Occasionally) • 1 (Very rarely) • 0 (Never)
22. **Do you say something and realize afterwards that it might be taken as insulting?**
    • 4 (Very often) • 3 (Quite often) • 2 (Occasionally) • 1 (Very rarely) • 0 (Never)
23. **Do you fail to hear people speaking to you when you are doing something else?**
    • 4 (Very often) • 3 (Quite often) • 2 (Occasionally) • 1 (Very rarely) • 0 (Never)
24. **Do you lose your temper and regret it?**
    • 4 (Very often) • 3 (Quite often) • 2 (Occasionally) • 1 (Very rarely) • 0 (Never)
25. **Do you leave important letters unanswered for days?**
    • 4 (Very often) • 3 (Quite often) • 2 (Occasionally) • 1 (Very rarely) • 0 (Never)
26. **Do you find you forget which way to turn on a road you know well but rarely use?**
    • 4 (Very often) • 3 (Quite often) • 2 (Occasionally) • 1 (Very rarely) • 0 (Never)
27. **Do you fail to see what you want in a supermarket (although it’s there)?**
    • 4 (Very often) • 3 (Quite often) • 2 (Occasionally) • 1 (Very rarely) • 0 (Never)
28. **Do you find yourself suddenly wondering whether you’ve used a word correctly?**
    • 4 (Very often) • 3 (Quite often) • 2 (Occasionally) • 1 (Very rarely) • 0 (Never)
29. **Do you have trouble making up your mind?**
    • 4 (Very often) • 3 (Quite often) • 2 (Occasionally) • 1 (Very rarely) • 0 (Never)
30. **Do you find you forget appointments?**
    • 4 (Very often) • 3 (Quite often) • 2 (Occasionally) • 1 (Very rarely) • 0 (Never)
31. **Do you forget where you put something like a newspaper or a book?**
    • 4 (Very often) • 3 (Quite often) • 2 (Occasionally) • 1 (Very rarely) • 0 (Never)
32. **Do you find you accidentally throw away the thing you want and keep what you meant to throw away – as in the example of throwing away the matchbox and putting the used match in your pocket?**
    • 4 (Very often) • 3 (Quite often) • 2 (Occasionally) • 1 (Very rarely) • 0 (Never)
33. **Do you daydream when you ought to be listening to something?**
    • 4 (Very often) • 3 (Quite often) • 2 (Occasionally) • 1 (Very rarely) • 0 (Never)
34. **Do you find you forget people’s names?**
    • 4 (Very often) • 3 (Quite often) • 2 (Occasionally) • 1 (Very rarely) • 0 (Never)
35. **Do you start doing one thing at home and get distracted into doing something else (unintentionally)?**
    • 4 (Very often) • 3 (Quite often) • 2 (Occasionally) • 1 (Very rarely) • 0 (Never)
36. **Do you find you can’t quite remember something although it’s “on the tip of your tongue”?**
    • 4 (Very often) • 3 (Quite often) • 2 (Occasionally) • 1 (Very rarely) • 0 (Never)
37. **Do you find you forget what you came to the shops to buy?**
    • 4 (Very often) • 3 (Quite often) • 2 (Occasionally) • 1 (Very rarely) • 0 (Never)
38. **Do you drop things?**
    • 4 (Very often) • 3 (Quite often) • 2 (Occasionally) • 1 (Very rarely) • 0 (Never)
39. **Do you find you can’t think of anything to say?**
    • 4 (Very often) • 3 (Quite often) • 2 (Occasionally) • 1 (Very rarely) • 0 (Never)
